# Author Correction: Staphylococcal superantigen-like protein 10 induces necroptosis through TNFR1 activation of RIPK3-dependent signal pathways

**DOI:** 10.1038/s42003-025-08379-z

**Published:** 2025-06-20

**Authors:** Nan Jia, Guo Li, Xing Wang, Qing Cao, Wanbiao Chen, Chengliang Wang, Ling Chen, Xiaoling Ma, Xuan Zhang, Yue Tao, Jianye Zang, Xi Mo, Jinfeng Hu

**Affiliations:** 1 Department of Clinical Laboratory, The First Affiliated Hospital of USTC, Division of Life Sciences and Medicine, University of Science and Technology of China, Hefei, 230026 Anhui China; 2 The Laboratory of Pediatric Infectious Diseases, Pediatric Translational Medicine Institute, Shanghai Children’s Medical Center, Shanghai Jiao Tong University School of Medicine, Shanghai, 200127 China; 3 Fujian Key Laboratory of Translational Research in Cancer and Neurodegenerative Diseases, Institute for Translational Medicine, School of Basic Medical Sciences, Fujian Medical University, Fuzhou, 350122 China; 4 Department of Laboratory Medicine, Shanghai Children’s Medical Center, Shanghai Jiao Tong University School of Medicine, Shanghai, 200127 China; 5 Department of Infectious Diseases, Shanghai Children’s Medical Center, Shanghai Jiao Tong University School of Medicine, Shanghai, 200127 China

**Keywords:** Pathogens, X-ray crystallography, Infectious diseases

Correction to: *Communications Biology* 10.1038/s42003-022-03752-8, published online 12 August 2022

In the version of the article initially published, due to figure preparation mistakes, there were presentation errors in Fig. 1b, where flow cytometry plots for SSL7-induced HEK293T cells were duplicated in Fig. 2d, and in Fig. 1c, where flow cytometry plots for Control, SSL7 and SSL10-induced HUVECs in Fig. 1c were duplicated in Supplementary Fig. 2a. Figure 1 and the corresponding Supplementary Data have been updated in the HTML and PDF versions of the article.

