# Supplementary material for: Staphylococcal superantigen-like protein 10 induces necroptosis through TNFR1 activation of RIPK3-dependent signal pathways
Source: Commun Biol. 2022 Aug 12;5:813. doi: 10.1038/s42003-022-03752-8 (PMC9374677; doi:10.1038/s42003-022-03752-8)
Supplement: Supplementary file 2 — Supplementary information [file 42003_2022_3752_MOESM2_ESM.pdf]

Supplementary Information: File contains supplementary methods, supplementary tables, supplementary figures and figures legends, figure exemplifying the gating strategy and unprocessed western blot images.

Supplementary Methods

Reagents

Cisplatin, IFN α-IFNAR-IN-1, and TAK-242 used to induce cells were purchased from MedChenExpress (New Jersey, USA).

Real-time Live-cell Analysis

HUVEC cells were seeded in 96-well plate (100 μL per well) at a density of 1×10^3 cells/well with or without 2 μM GFP-SSL10 or GFP treatment. The fluorescence produced by GFP was then observed with live-cell dynamic imaging and analysis system (Incucyte S3, Essen Bioscience, USA).

Laser Scanning Confocal Microscope

HUVEC cells were seeded on coverslips in a 24-well plate and then incubated for 4 h before being incubated with 2 μM GFP-SSL10 protein resuspended in Opti-MEM medium for 20 min. After treatment, the cells were washed twice with PBS to remove unbound proteins and then fixed with 4% paraformaldehyde for 20 min. After fixation, cells were washed three times with PBS and then incubated with DAPI. After three additional PBS washes, coverslips were mounted onto slides using antifade mounting medium (Beyotime, Shanghai, China). The cells were visualized using a laser scanning confocal microscope (Leica, Wetzlar, Germany).

Protein Expression and Purification

DNA fragments encoding amino acid residues 31-356 of SSL3, 31-232 of SSL8, and 31-227 of SSL11 were amplified by PCR from *S. aureus* strain Mu50 and cloned into the pET-22b (+) vector with a N-terminal 6 × His tag. SSL3, SSL8, and SSL11 were expressed in *E. coli* BL21 (DE3) and induced with 0.4 mM IPTG for 20 h at 16 ℃ when OD_{600} reached 0.6. These proteins were purified as recombinant SSL10.

GFP-SSL10 used for laser scanning confocal microscopy was cloned into the pET-28a (+) vector (Novagen) with the GFP- and 6 × His-tag fused to its N-terminus. The protein was expressed and purified as recombinant SSL10.

DNA fragment of human prothrombin Gla domain (amino acid residues 44-89) was amplified by PCR and cloned into the pGEX-6P-1 vector with an N-terminal GST-tag and a 9 × (GGS)-linker prior to the target fragment. The recombinant protein GST-Gla was expressed in *E. coli* Rosetta2 (DE3) strain and purified by GST-affinity chromatography.

Patients

A total of 16 patients who were positive for *S. aureus* in their blood culture were recruited in Shanghai Children's Medical Center (SCMC) from December 2018 to December 2019. According to the Third International Consensus Definitions for Sepsis and Septic Shock (Sepsis-3)[1], six patients were with septic shock, and eight patients had multiple organ dysfunction syndrome (MODS). In addition, ten patients had hypoproteinemia, who had plasma albumin concentration less than 30 g/L[2]. Hypoproteinemia, septic shock or MODS was diagnosed by a senior clinician blinded

to the *ssl7* or *ssl10* level detection.

The study was approved by the Institutional Review Board and the Ethics Committee of Shanghai Children's Medical Center (SCMCIRB-W2020054), and formal written informed consent was assigned by each patient or their parents.

Quantitative Real-Time PCR (qRT-PCR)

S. *aureus* strains isolated from the patients were cultured in TSB medium, and the cells were collected and processed with 1 mL of RNAiso Plus (TaKaRa) in combination with 0.1-mm-diameter-silica beads in a FastPrep-24 automated system (MP biomedicals Solon, OH, United States), and then total RNA was isolated with RNeasy Mini Kit (QIAGEN, Hilden, Germany). cDNA was synthesized using a PrimeScript^{TM} RT Master Mix (TaKaRa Bio Inc., Shiga, Japan). Quantitative real-time PCR was performed using a Hieff qRCP SYBR Green Master Mix (Yeason, Shanghai, China) and CFX Connect Real-Time System (BIO-RAD). Expression of *ssl7* and *ssl10* gene in the *S. aureus* strains was normalized to the level of *gyrB* from the sample and was analyzed by $2^{-\Delta Ct}$. When the *ssl10* or *ssl7* gene expression cannot be detected, the cycle threshold (Ct) value of the sample was calculated as 40. Primers used were as follows: 5'-ACAGCGTAGTTATGAGGGGTTA-3' and 5'-ATTGGTGCACTGACAACTCC-3' for *ssl10*, 5'-CGGTTCTAACGTTGTACGCT-3' and 5'-ACCACAAAGACATTCT GGCCT-3' for *ssl7*, and 5'-CAAATGATCACAGCATTTGGTACAG-3' and 5'-CGG CATCAGTCATAATGACGAT-3' for *gyrB*. Comparisons of the *ssl10* and *ssl7* levels between the two groups of patients were analyzed by the nonparametric Mann-

Whitney test using GraphPad Prism Version 6.0 software program. p values < 0.05 were considered statistically significant.

Supplementary Tables

Supplementary Table 1. The docking scores and binding free energies of the top 10 predicted SSL10/TNFR1^{ECD} models generated by HawkDock.

Model	Docking score	Binding free energy (kcal/mol)
1	-5383.28	-13.16
2	-4934.31	-22.02
3	-4780.94	-7.51
4	-4340.73	-22.34
5	-4325.42	-37.63
6	-4241.46	-14.11
7	-4039.77	-11.18
8	-4001.47	-22.29
9	-3967.16	-30.28
10	-3959.68	-28.98

Supplementary Table 2. Mutated residues in SSL10 mutants constructed according to the predicted SSL10/TNFR1^{ECD} model 1, 2, 4 and 8.

SSL10 Mutants	Mutated residues
M1	H64D, N67R, N68K, R70E, K106D, K206D, F207E, K208E, Y209R
M2	K52E, T53S, M54F, E55K, R74E, I76Q, K166E, K169E
M4	R74E, K147E, Y149D, Y152L, K154D, K166E, K169E, H170D, E173K
M8	N67R, N68K, Q79K, K87E, K106E, Y114S, K204E, K208E

Supplementary Figures:

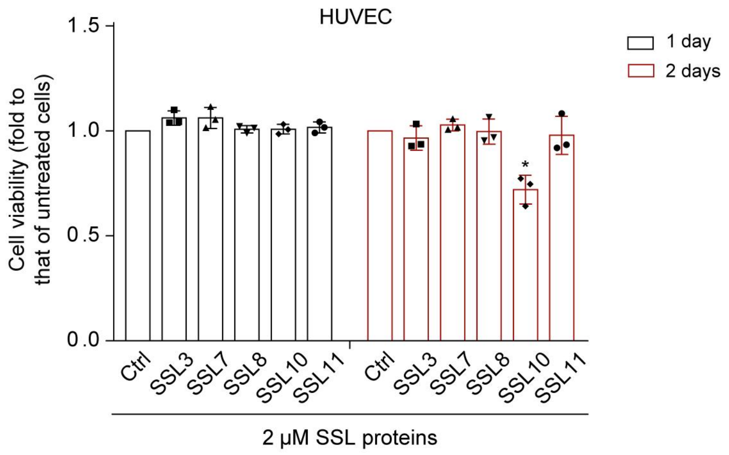

Supplementary Figure 1. Cytotoxicity of different SSL proteins on HUVEC. Cell viability of HUVEC cells treated with 2 μM SSL3, SSL7, SSL8, SSL10, or SSL11 for 24 h or 48 h was detected by MTS assay, and was then normalized to that of the untreated cells (*i.e.*, Ctrl cells, buffer treated cells) on the same day. All data represent mean ± SD from three independent experiments. *, $p < 0.05$ compared to the Ctrl cells (buffer-treated cells) by two-way ANOVA.

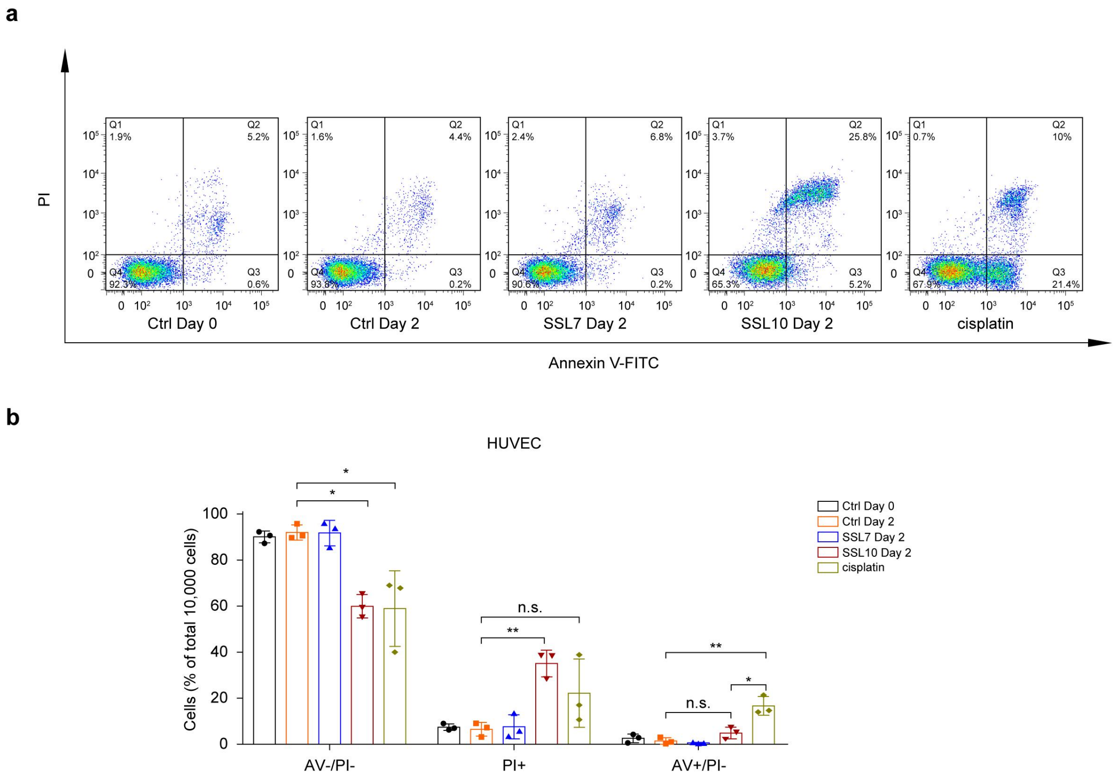

Supplementary Figure 2. SSL10-treated cells undergo necrosis. HUVEC were treated with buffer, SSL7 or SSL10 for 48 h. HUVEC cells before treatment and cells treated with cisplatin for 24 h were used as a negative control and a positive control for cellular apoptosis, respectively. All cells were detected by flow cytometry after Annexin V/PI staining. The dot plot (**a**) is representative of three independent experiments, and the quantification results are shown as a bar graph (**b**). All data represent mean ± SD from three independent experiments. *, $p < 0.05$; **, $p < 0.01$; n.s., not significant as indicated by one-way ANOVA.

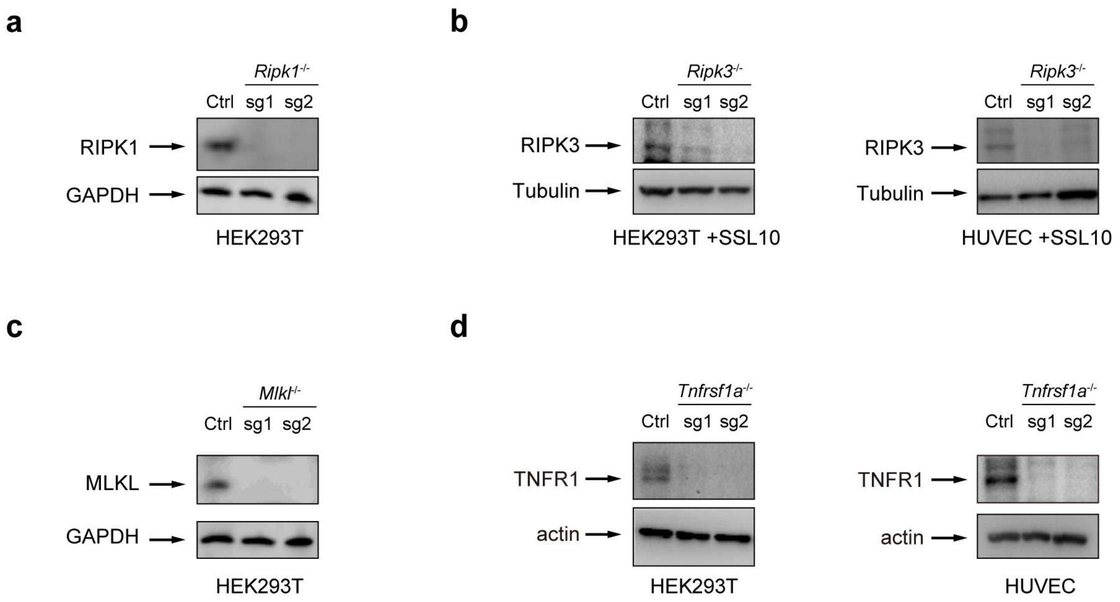

Supplementary Figure 3. Verification of RIPK1, RIPK3, MLKL, and TNFR1 knockout in HEK293T and HUVEC cells. (**a**, **c**) RIPK1 (**a**) and MLKL (**c**) were detected by immunoblotting in the whole cell lysates of WT and knockout HEK293T cells. (**b**) RIPK3 knockout in HEK293T and HUVEC cells were confirmed after SSL10 treatment. (**d**) TNFR1 was detected by immunoblotting in the whole cell lysates of WT and knockout HEK293T and HUVEC cells.

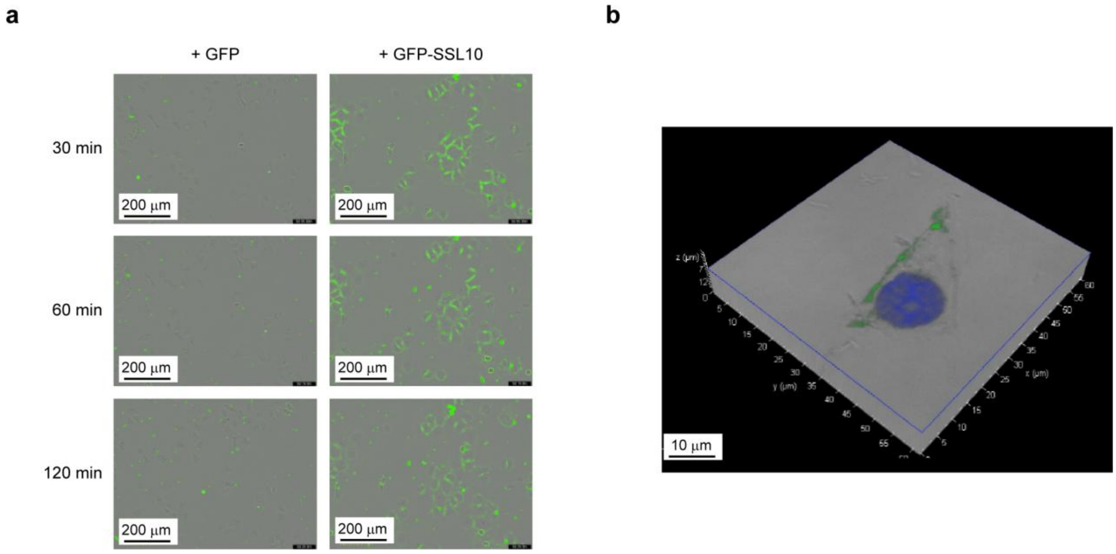

Supplementary Figure 4. Binding of GFP-SSL10 to the cell surface of HUVEC. (**a**) Real-time live-cell analysis of HUVEC incubated with GFP-SSL10 or GFP via the IncuCyte® S3 Live-Cell Analysis System. (**b**) HUVEC were incubated with GFP-SSL10 for 30 min and then observed with laser scanning confocal microscopy.

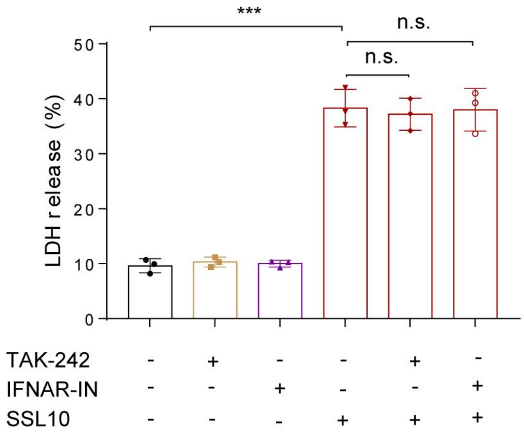

Supplementary Figure 5. Necroptosis induced by SSL10 is not dependent on TLR4 or IFNAR1. HEK293T cells were pretreated with 10 μM TAK-242 or 1 μM IFN alpha-IFNAR-IN-1 (IFNAR-IN) for 1 h followed by SSL10 treatment for 48 h, and the LDH release was detected. All data are presented as the mean ± SD from three independent experiments. ***, $p < 0.001$ compared to the Ctrl cells (buffer-treated cells); n.s., not significant, by one-way ANOVA.

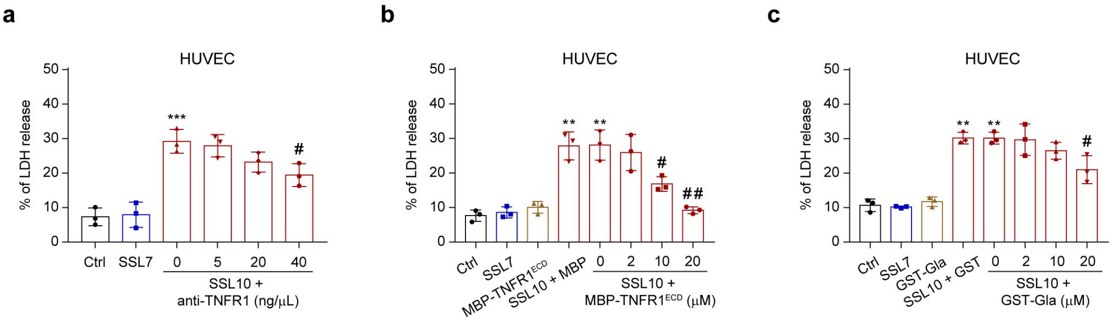

Supplementary Figure 6. Competitive inhibition of SSL10-induced cytotoxicity. HUVEC cells were pretreated with anti-TNFR1 antibody (**a**), purified MBP-TNFR1^{ECD} (**b**) or GST-Gla (**c**) at different doses, and then treated with 2 μM SSL10 as indicated. The LDH release was detected. Cells treated with 2 μM SSL7 were used as a negative protein control throughout the experiments. All data represent mean ± SD from three independent experiments. **, $p < 0.01$; ***, $p < 0.001$ compared to the Ctrl cells (buffer-treated cells). #, $p < 0.05$; ##, $p < 0.01$ compared to the SSL10-treated cells by one-way ANOVA.

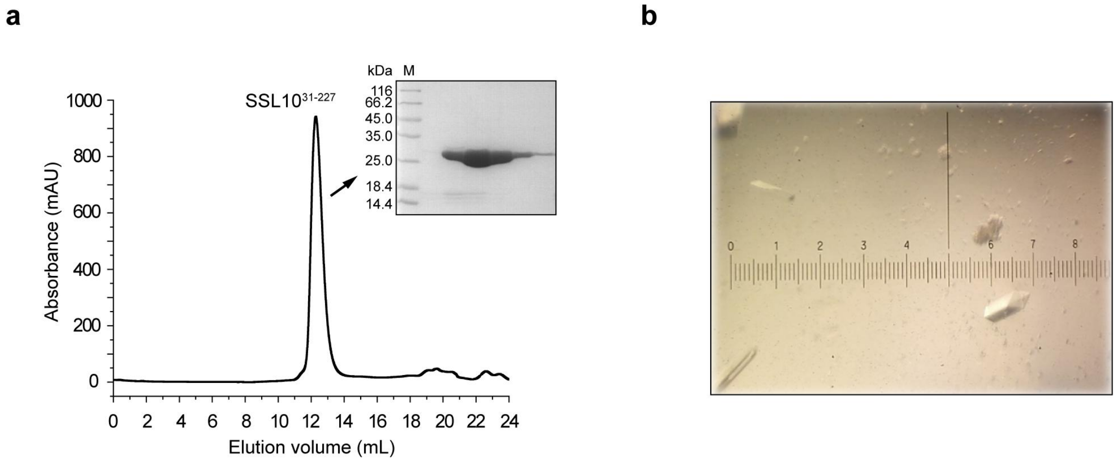

Supplementary Figure 7. Purification and crystallization of SSL10. (**a**) Gel filtration of SSL10 with Superdex75 (10/300) column (GE Healthcare). The eluent is analyzed by SDS-PAGE. (**b**) Crystals of SSL10 under the condition of 2.1 M DL-Malic acid, pH 7.0.

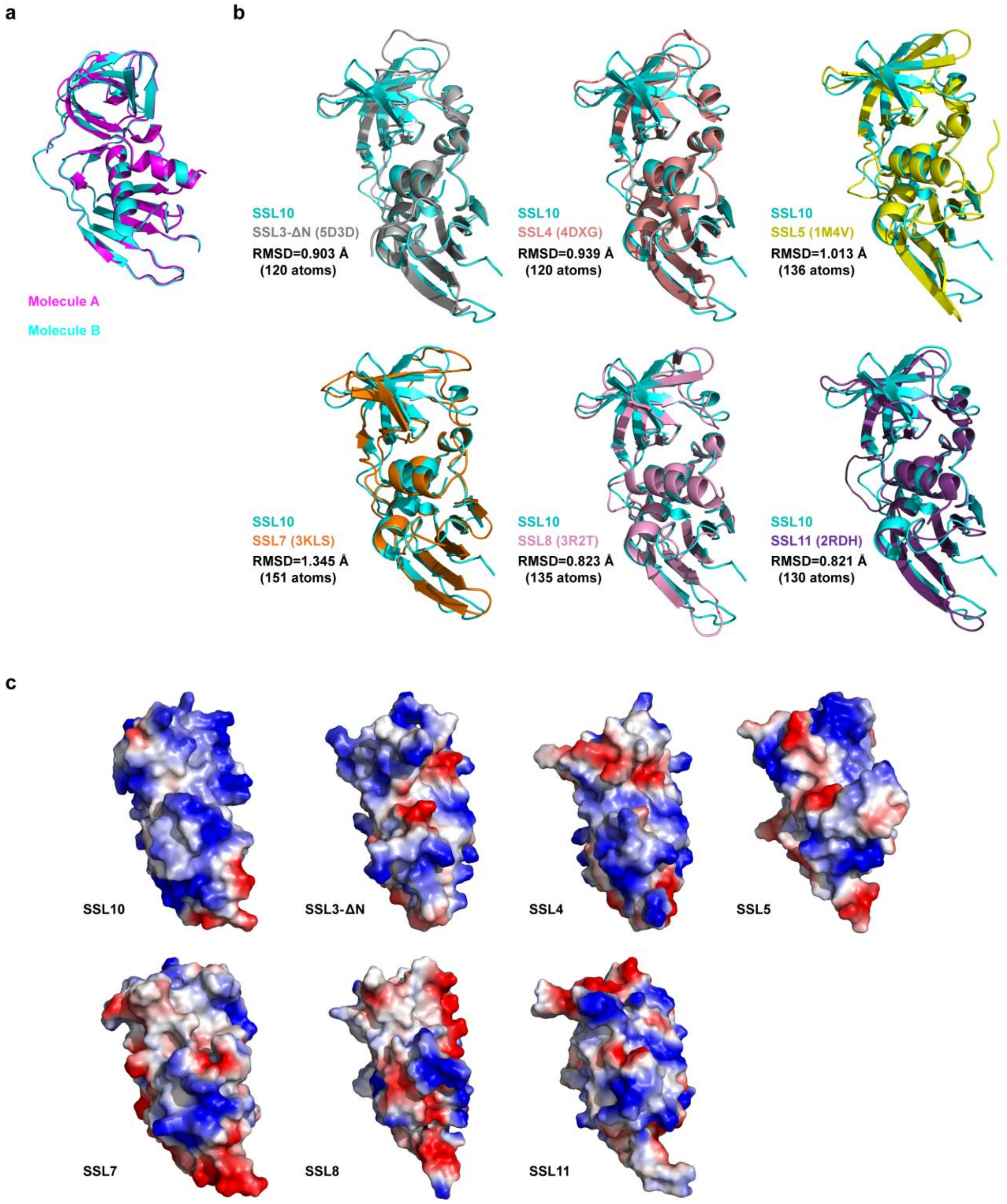

Supplementary Figure 8. Structural comparison of SSL10 with other SSL proteins. (**a**) Structure superposition of the two SSL10 molecules from one asymmetric unit. Molecular A and B is colored magenta and cyan, respectively. (**b**) Structure comparison of SSL10 (cyan) with SSL3ΔN (gray, PDB ID: 5D3D), SSL4 (deepsalmon, PDB ID: 4DXG), SSL5 (yellow, PDB ID: 1M4V), SSL7 (orange, PDB ID: 3KLS), SSL8 (pink, PDB ID: 3R2T), and SSL11 (purple, PDB ID: 2RDH),

respectively. The RMSD value of each comparison are indicated. (**c**) The electrostatic surface potentials of SSL10, SSL3ΔN, SSL4, SSL5, SSL7, SSL8, and SSL11.

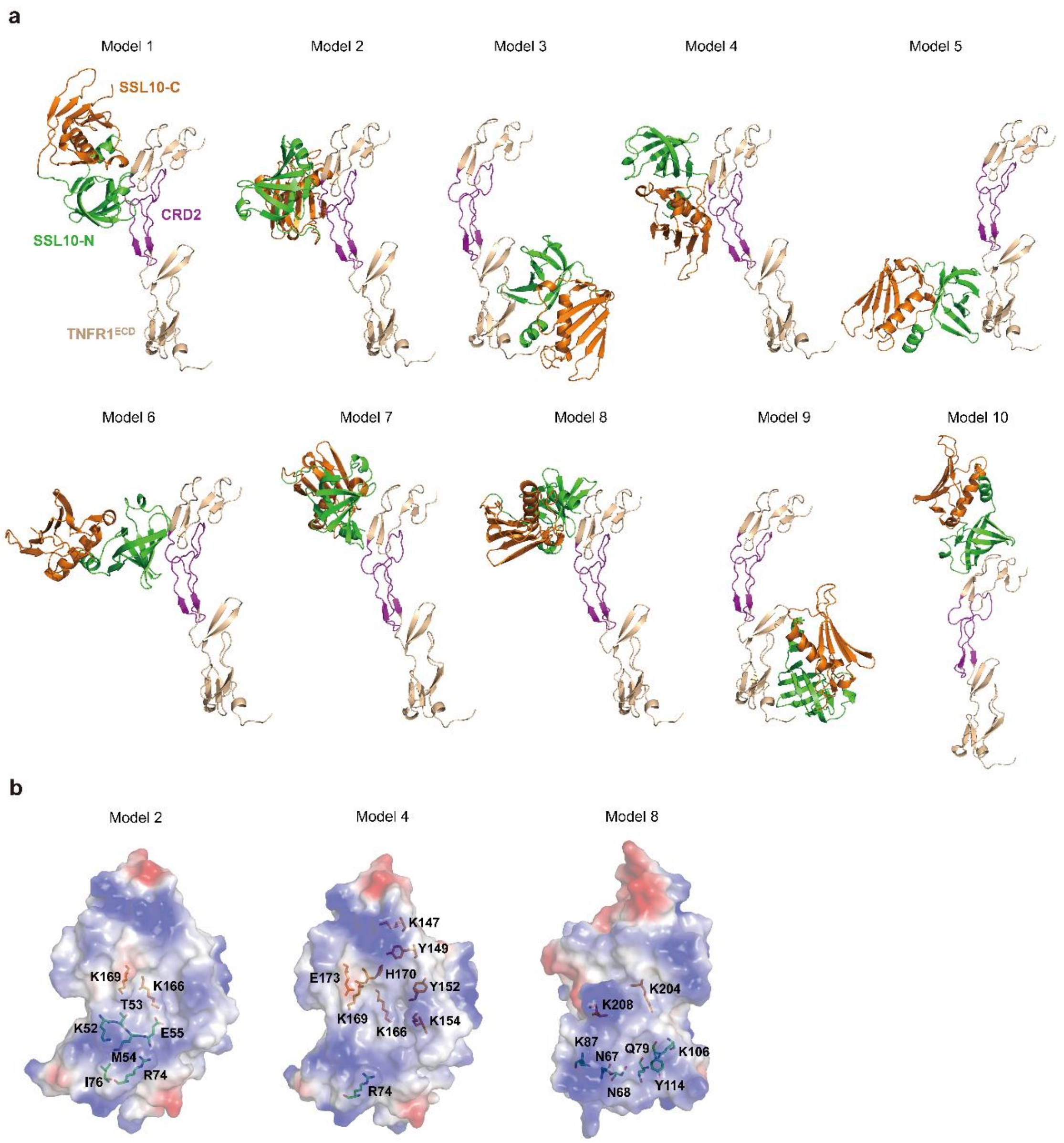

Supplementary Figure 9. The top 10 SSL10/TNFR1^{ECD} complex models generated by HawkDock webserver. (**a**) Cartoon presentation of SSL10/TNFR1^{ECD} models. The N- terminal domain of SSL10, the C- terminal domain of SSL10, the CRD2 region of TNFR1^{ECD}, and the other regions of TNFR1^{ECD} being colored in green, orange, magenta, and light brown, respectively. (**b**) The electrostatic surface potentials of SSL10 molecules from Model 2, 4, and 8. The interface residues on SSL10 responsible for TNFR1 binding are shown as sticks and labeled in black. The positive and negative charge are colored blue and red, respectively.

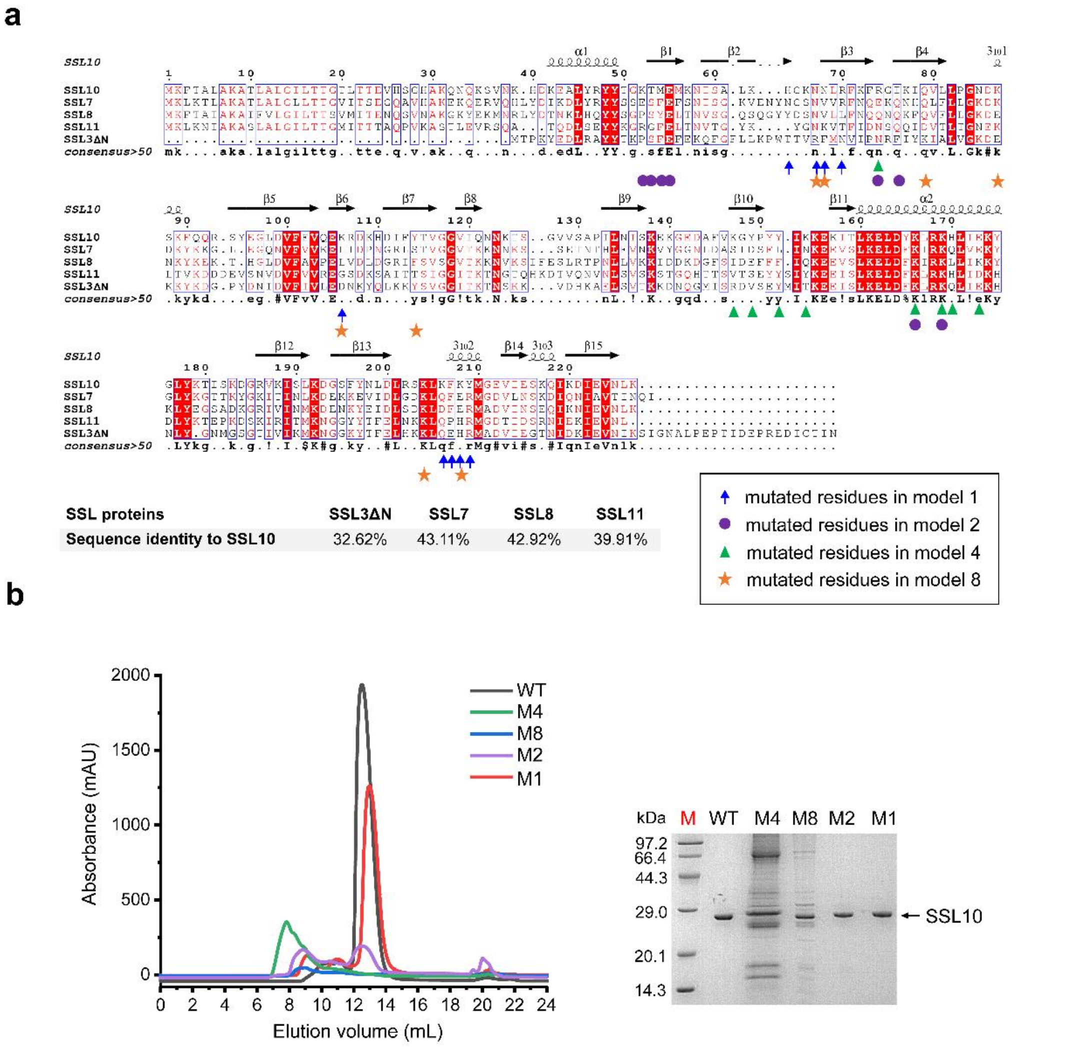

Supplementary Figure 10. Purification of SSL10 mutants. (**a**) Multiple sequence alignment of SSL10 with SSL3ΔN, SSL7, SSL8, and SSL11 generated by MultAlin and ESPript. The secondary structure elements of SSL10 are shown above the sequences. Sequence identities of SSL3ΔN, SSL7, SSL8, and SSL11 to SSL10 are calculated by Clustal Omega and shown at the bottom. SSL10 residues desired for mutation for Model 1, 2, 4, and 8 are indicated by blue arrows, purple circles, green triangles, and orange stars, respectively. (**b**) Gel filtration of wild type and mutant SSL10 (M1, M2, M4, and M8) by using the Superdex75 (10/300) column (GE Healthcare). The purified proteins were verified by SDS-PAGE.

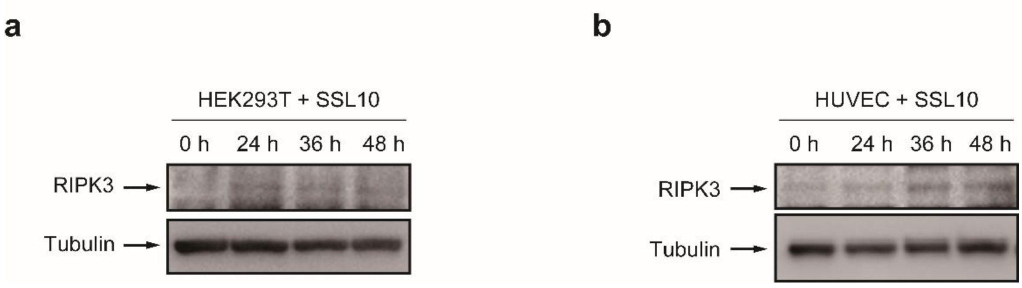

Supplementary Figure 11. SSL10 induces an increase in the protein level of RIPK3. RIPK3 was detected by immunoblotting in the whole cell lysates of HEK293T (**a**) and HUVEC (**b**) cells treated with 2 μM SSL10 for 0, 24, 36, or 48 h.

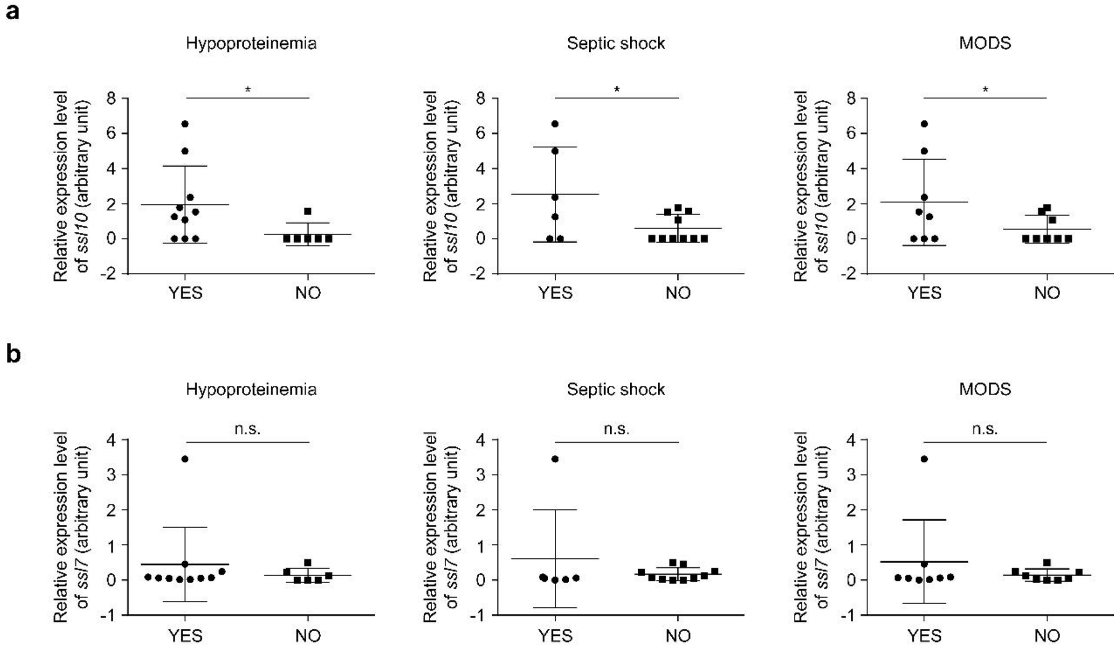

Supplementary Figure 12. mRNA levels of *ssl10* and *ssl7* in *S. aureus* strains. The gene expression levels of *ssl10* (**a**) and *ssl7* (**b**) isolated from the blood cultures of sixteen children infected by *S. aureus* were determined by real-time qPCR. The data were analyzed by the nonparametric Mann-Whitney test using GraphPad Prism Version 6.0 software program. *, $p < 0.05$; n.s., not significant.

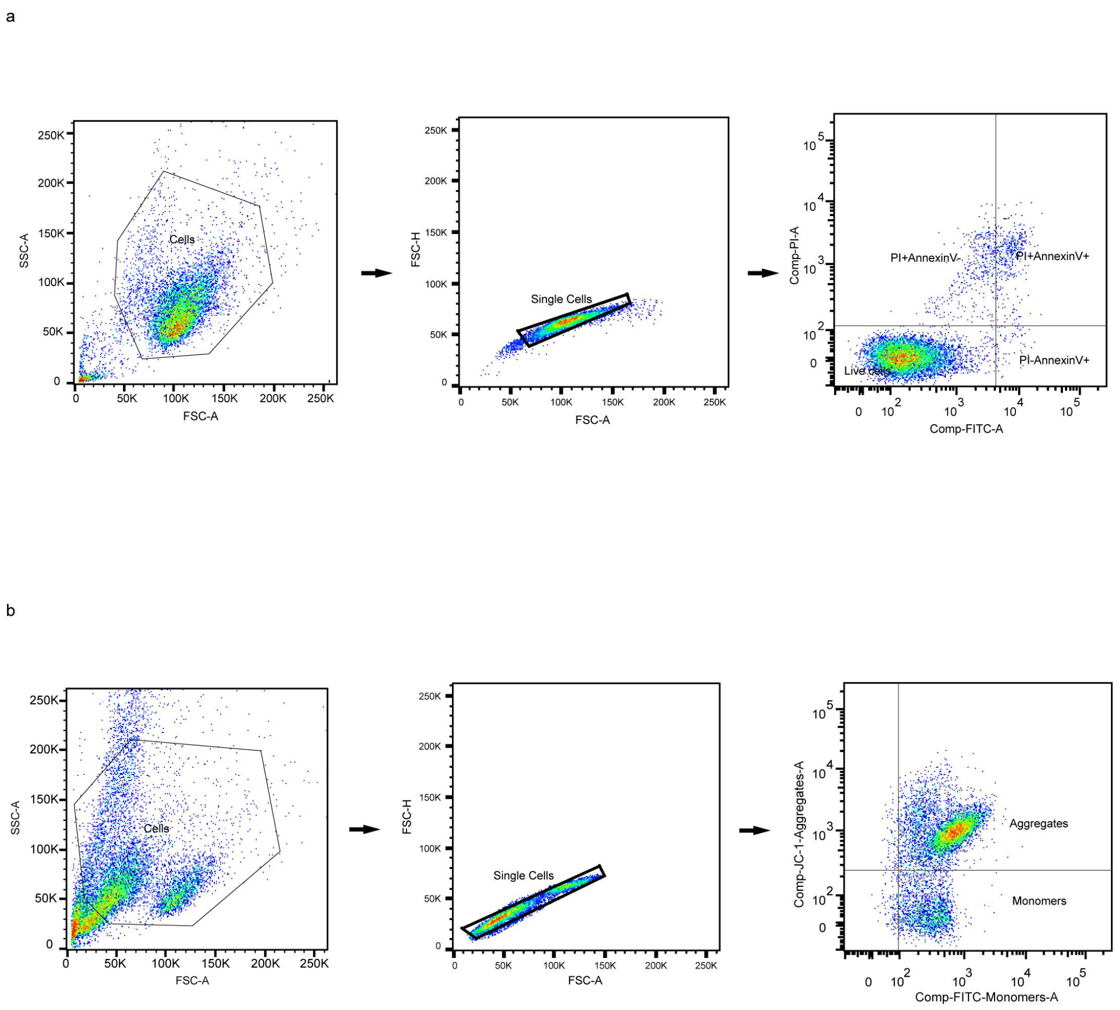

Supplementary Figure 13. Flow cytometry gating strategy. (a) For cell viability analysis, a cell gate was selected in a SSC-A versus FSC-A plot, followed by a gating on singlets in a FSC-H versus FSC-A plot. Cells were further plotted as PI versus AnnexinV-FITC to allow identification of necrosis or apoptosis cells. (b) For the analysis of depolarization of the mitochondrial membrane, a cell gate was selected in a SSC-A versus FSC-A plot, followed by a gating on singlets in a FSC-H versus FSC-A plot. Cells were further plotted JC-1 aggregates (PI channel) versus JC-1 monomers (FITC channel) to allow identification of mitochondrial depolarization.

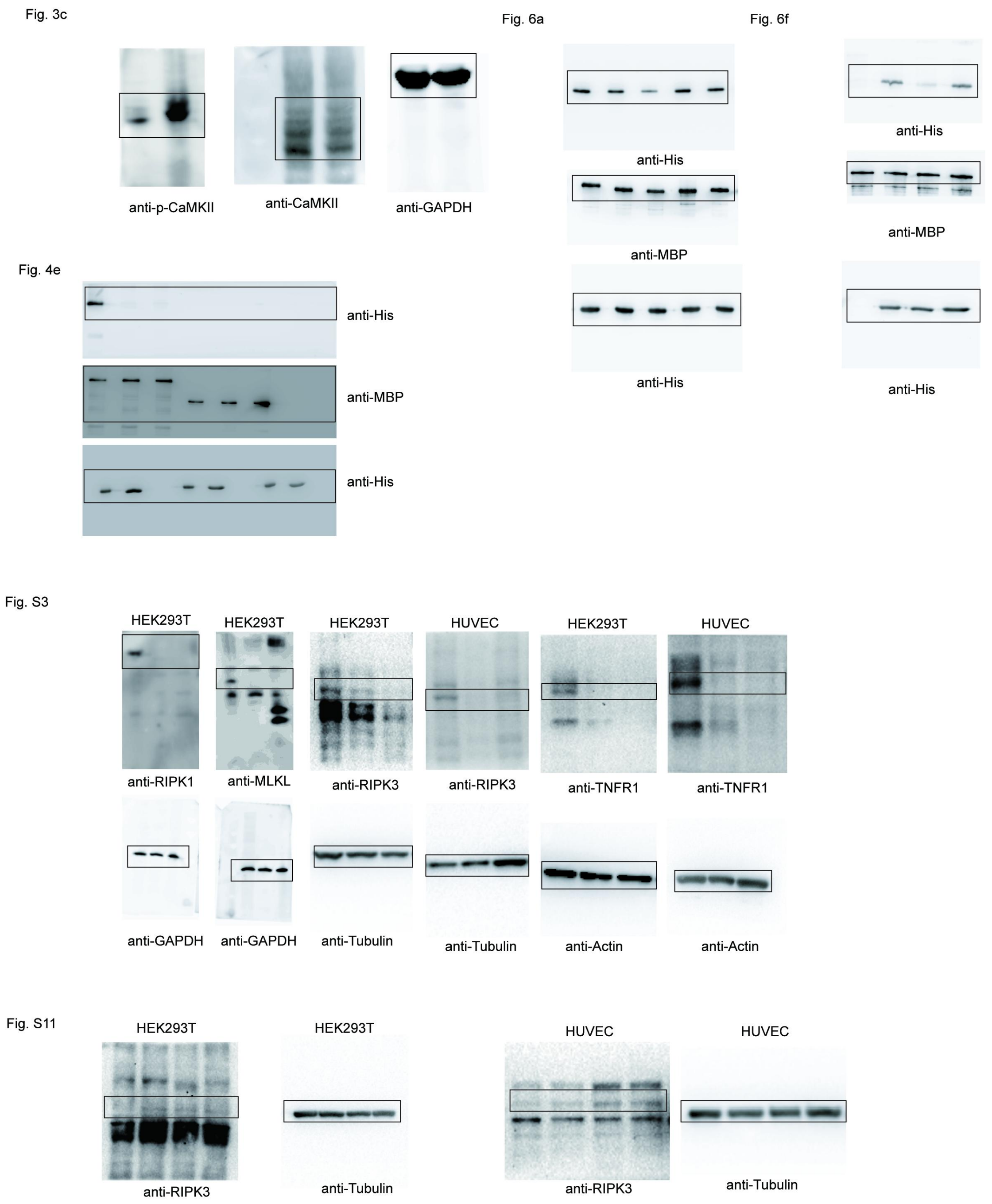

Supplementary Figure 14. Unprocessed western blot images.

Supplementary References:

1 Shankar-Hari, M. *et al.* Developing a New Definition and Assessing New Clinical Criteria for Septic Shock: For the Third International Consensus Definitions for Sepsis and Septic Shock (Sepsis-3). *JAMA* **315**, 775-787, doi:10.1001/jama.2016.0289 (2016).

2 Furukawa, M., Kinoshita, K., Yamaguchi, J., Hori, S. & Sakurai, A. Sepsis patients with complication of hypoglycemia and hypoalbuminemia are an early and easy identification of high mortality risk. *Intern Emerg Med* **14**, 539-548, doi:10.1007/s11739-019-02034-2 (2019).
