# Supplementary material for: Staphylococcal superantigen-like protein 10 induces necroptosis through TNFR1 activation of RIPK3-dependent signal pathways
Source: Commun Biol. 2022 Aug 12;5:813. doi: 10.1038/s42003-022-03752-8 (PMC9374677; doi:10.1038/s42003-022-03752-8)
Supplement: Supplementary file 3 — Description of Additional Supplementary Files [file 42003_2022_3752_MOESM3_ESM.pdf]

Description of Additional Supplementary Files

File name: Supplementary Data 1
Description: The source data behind the graphs and charts in the paper.
